# Intimate partner violence against women in Maputo city, Mozambique

**DOI:** 10.1186/1472-698X-12-35

**Published:** 2012-12-14

**Authors:** Antonio Eugenio Zacarias, Gloria Macassa, Leif Svanström, Joaquim JF Soares, Diddy Antai

**Affiliations:** 1 Department of Public Health Sciences, Division of Social Medicine, Karolinska Institutet, Stockholm SE-17176, Sweden; 2 Eduardo Mondlane University, Faculty of Medicine, Maputo City, Mozambique; 3 Department of Occupational and Public Health Sciences, University of Gävle, Gävle, Sweden; 4 Department of Health Sciences, Division of Public Health Sciences, Mid Sweden University, Sundsvall, Sweden; 5 Center for Global & Population Health, The Angels Trust Nigeria, Abuja, Nigeria

**Keywords:** Intimate partner violence, Victims, Controlling behaviours, Perpetration, Abuse as a child

## Abstract

**Background:**

There is limited research about IPV against women and associated factors in Sub-Saharan Africa, not least Mozambique. The objective of this study was to examine the occurrence, severity, chronicity and “predictors” of IPV against women in Maputo City (Mozambique).

**Methods:**

Data were collected during a 12 month-period (consecutive cases, with each woman seen only once) from 1,442 women aged 15–49 years old seeking help for abuse by an intimate partner at the Forensic Services at the Maputo Central Hospital, Maputo City, Mozambique. Interviews were conducted by trained female interviewers, and data collected included demographics and lifestyle variables, violence (using the previously validated Revised Conflict Tactics Scale (CTS2), and control (using the Controlling Behaviour Scale Revised (CBS-R). The data were analysed using bivariate and multivariate methods.

**Results:**

The overall experienced IPV during the past 12 months across severity (one or more types, minor and severe) was 70.2% (chronicity, 85.8 ± 120.9).^a^ Severe IPV varied between 26.3-45.9% and chronicity between 3.1 ± 9.1-12.8 ± 26.9, depending on IPV type. Severity and chronicity figures were higher in psychological aggression than in the other IPV types. Further, 26.8% (chronicity, 55.3 ± 117.6) of women experienced all IPV types across severity. The experience of other composite IPV types across severity (4 combinations of 3 types of IPV) varied between 27.1-42.6% and chronicity between 35.7 ± 80.3-64.9 ± 110.9, depending on the type of combination. The combination psychological aggression, physical assault and sexual coercion had the highest figures compared with the other combinations. The multiple regressions showed that controlling behaviours, own perpetration and co-occurring victimization were more important in “explaining” the experience of IPV than other variables (e.g. abuse as a child).

**Conclusions:**

In our study, controlling behaviours over/by partner, own perpetration, co-occurring victimization and childhood abuse were more important factors in “explaining” sustained IPV. More investigation into women’s IPV exposure and its “predictors” is warranted in Sub-Saharan Africa, particularly Mozambique.

## Background

Intimate partner violence (IPV) towards women is an enduring public health problem world-wide, with effects ranging from financial hardships and decreased intimacy to high rates of morbidity and mortality. In sub-Saharan Africa (SSA), the poor living and health conditions of women may be further exacerbated by IPV [1,2]. The Centre of Disease and Prevention (http://www.cdc.gov/) and Saltzman et al. (2002) [3] define IPV as “physical, sexual or psychological harm by current or former partner or spouse” [3]. Data from SSA show that 5–29% of women are physically assaulted by their male intimate partners each year and 13–59% during life-time, with the annual rates of assault with injury ranging from 2–45.9% [1,2,4-10]. The 12-month sexual coercion rates vary between 9.1 - 44.4% yearly and ever coerced between 16.5 - 58.6%, [1,2,5-7,10,11], whilst verbal/emotional aggression during the past 12 months and life-time may be as elevated as 51% and 67.5%, respectively [5,6,10,12-14]. Different types of IPV often co-occur, and a review of IPV in 7 African countries (Cameron, Kenya, Malawi, Rwanda, Uganda, Zambia and Zimbabwe), showed that rates of co-occurring emotional aggression, physical assault and sexual coercion varied between 3.6 - 8.3%, and physical assault and sexual coercion between 6.8 - 30.1% [5].

Male coercion or controlling behaviour (e.g. my partner always wants to know where I am) has been associated with high IPV risk for women [2,5,14-16], but in certain conditions (e.g. women’s unfaithfulness) both men and women may condone abuse [12,17]. Controlling behaviour in intimate relationships has been described as “when one partner (most commonly the man) uses threats and emotional abuse to maintain control over the other partner (most commonly the woman)” [18]. Controlling behaviour reflects a power structure within the relationship, and has been shown to be significantly associated with higher likelihood of use of violence [16], by men against women. A recent study with university students from Ghana showed that the frequency of use of controlling behaviours and victimization/perpetration amongst men and women (e.g. control the other’s money) were similar, and that controlling behaviours were a “predictor” of IPV [19]. Women’s experiences of abuse during childhood in SSA may be relatively common [1,8,20-24]; however, few studies seem to have addressed the relation between abuse during childhood and IPV exposure in adulthood, with results showing a positive association [8,24]. Data on the relation between IPV and socio-economic status are conflicting, with some studies reporting that women living in poor socio-economic conditions (e.g. unemployed) are more exposed to IPV [8,25], whilst others suggest that empowered women (e.g. employed) are at higher risk [13,15]. Heavy alcohol use by men and women has also been associated with IPV [8,12,26,27]. The influence of women’s IPV perpetration on their own victimization in SSA has attracted little attention, but studies indicate that women perpetrate abuse against their male intimate partners at rates from 0.5–27% [2,10,12-15,26,28]. Two studies in Mozambique reported that 8% [25] and 37% [29] of men, respectively, were physically assaulted by their female intimate partners during the past 12 months.

Few studies have addressed the association between controlling behaviours and women’s exposure to IPV in Sub-Saharan Africa, and only one study has examined the connection between controlling behaviours and victimization/perpetration among both sexes [19]. Evidence suggests also that women`s use of coercion is related to their IPV victimization/perpetration [19]; such knowledge may be important to tailor specific interventions to deal with the influence of control in violence, but also to deal with dominance issues in general. Thus, to fill this gap, the current study investigated the frequency, severity and chronicity of IPV (psychological aggression, physical assault, sexual coercion, physical assault with injury) experienced by women seeking help for abuse by partner using the Conflict Tactics Scales 2 (CTS2) [30], as well as the help-seeking women’s experiences of being controlled and using controlling behaviours in relation to their IPV exposure using the Controlling Behaviour Scale Revised (CBS-R) [31,32].

### Conceptual framework

Feminist theory argues that the risk factors of IPV are at socio-political levels. This theory looks at how men and women are acculturated into protagonists of power/ controlling behaviours [33-35]. This framework conceptualizes that power imbalances within the patriarchal societies create a gender social order that gives men rights and authority within the family/relationship over women. This results in men exercising power and control over women in several ways including the use violence/abuse as a tool to keep power/controlling behaviour [33-35]. This theory offers a comprehensive understanding of factors that may influence IPV exposure. The approach used reflect that causes and outcomes of IPV due the protagonist of power/controlling behaviours surrounded by the relationship/family associated with demographic, socio-economic and life-style factors.

The aim of this study was therefore to: (i) describe women’s experiences of IPV by type (psychological aggression, physical assault, sexual coercion, physical assault with injury), overall IPV and co-occurring IPV during the past 12 months; (ii) examine the association between IPV types, and demographic, socio-economic and life-style variables; and (iii) Identify and quantify factors associated with IPV by type (e.g. controlling behaviours).

It is hypothesized that the traditional gender role associated with demographic/socio-economic factors and controlling behaviours by partner increase the likelihood of being victimized of IPV.

## Methods

### Participants/Setting

A total of 1,500 women (aged 15–49 years) living in Maputo City, Mozambique were recruited to participate in this study (from a total population of 424,194 women within this age group in Maputo City). The women contacted the Forensic Services at the Maputo Central Hospital for abuse by partner, and the data was collected during one year (consecutive cases). Of the total number of women recruited, 1,442 (96.1%) answered the questionnaires and 58 (3.9%) refused to answer. Of the total number of women who answered the questionnaires (i.e. 1,442), 1433 answered questions on psychological aggression and sexual coercion, whilst 1432 answered questions on physical assault with and without injury, respectively (Table 1).

**Table 1:** Demographic/socio-economic and life-style characteristics of women as victims of IPV during the past 12 months

**Variables**	**Psychological aggression (n = 1433)**				**Physical assault (n = 1432)**			
	**Yes**		**No**		**Yes**		**No**	
	**n**	**%**	**n**	**%**	**n**	**%**	**n**	**%**
**Age** (years) (n)	NS				NS			
Mean ± SD	28.7 ± 8.1	28.8 ± 8.7			28.7 ± 7.9		29.5 ± 8.8	
**Marital status** (n)	(1405)		χ^2^(3) = 51.7, p < 0.0001		(1404)		χ^2^(3) = 22.6, p < 0.0001	
Single	489	53.5	251	51.2	388	51.1	352	54.6
Married/cohabitant	302	33	103	21	253	33.4	151	23.4
Divorced/separated	88	9.6	90	18.4	86	11.3	92	14.3
Widow	36	3.9	46	9.4	32	4.2	50	7.7
**Children at home **(n)	(1348)		NS		(1347)		NS	
Yes	649	73.1	352	76.5	554	75	446	73.4
**Housing **(n)	(1408)		NS		(1407)		NS	
Conventional^a^	787	85.5	411	84.4	650	84.8	547	85.3
Non-conventional^b^	134	14.5	76	15.6	116	15.2	94	14.7
**Education **(n)	(1423)		χ^2^(3) = 17.2, p = 0.0006		(1422)		NS	
No^c^	45	4.8	46	9.4	43	5.5	48	7.4
Low^d^	236	25.4	148	30.1	206	26.6	178	27.5
Inter^e^	519	55.7	241	49	427	55.2	333	51.4
* High*^*f*^	131	14.1	57	11.5	98	12.7	89	13.7
**Occupational status **(n)	(1381)		NS		(1380)		NS	
Blue-collar worker	504	55.8	294	61.5	440	58.8	358	56.7
Low white-collar worker	47	5.2	18	3.8	35	4.7	30	4.7
Middle/high white-collar worker	103	11.4	50	10.5	86	11.5	67	10.6
Student/other	249	27.6	116	24.2	187	25	177	28
**Socio-economic status **(n)	(1417)		NS		(1416)		NS	
Work for others^g^	320	34.4	145	29.8	277	35.8	188	29.3
Liberal/own business^h^	149	16	99	20.3	124	16	124	19.3
Student	251	27	115	23.6	190	24.6	175	27.3
Domestic/other^i^	210	22.6	128	26.3	183	23.6	155	24.1
**Salary/financial resources **(n)	(1386)		NS		(1386)		NS	
Yes	424	46.4	213	45.1	362	47.5	275	44.1
**Financial strain **(n)	(1405)		NS		(1404)		NS	
Yes	774	83.9	420	87.1	651	84.8	542	85.2
**BMI **(n)	(1316)		NS		(1315)		NS	
Mean ± SD	25 ± 4.6		24.5 ± 4.8		25.1 ± 4.6		24.5 ± 4.8	
**Alcohol use **(n)	(1412)		χ^2^(1) = 8, p = 0.0046		(1411)		χ^2^(1) = 9.4, p = 0.0021	
Yes	364	39.5	156	31.8	310	40.5	210	32.6
**Cigarette use **(n)	(1399)		NS		(1398)		NS	
Yes	65	7.1	33	6.8	60	7.9	38	5.9
**Age **(years) (n)	NS				NS			
Mean ± SD	28.6 ± 7.9		29.6 ± 8.7		29.6 ± 7.7		28.8 ± 8.7	
**Marital status **(n)	(1405)		χ^2^(3) = 30.1, p < 0.0001		(1404)		χ^2^(3) = 32.8, p < 0.0001	
Single	390	54.6	350	50.7	209	43.5	531	57.5
Married/cohabitant	230	32.2	175	25.3	174	36.3	230	24.9
Divorced/separated	68	9.5	110	15.9	75	15.6	103	11.1
Widow	26	3.7	56	8.1	22	4.6	60	6.5
**Children at home **(n)	(1348)		NS		(1347)		χ^2^(1) = 42.1, p < 0.0001	
Yes	513	74.2	488	74.3	397	84.8	603	68.6
**Housing **(n)	(1408)		NS		(1407)		χ^2^(1) = 6.6, p = 0.0103	
Conventional^a^	615	85.5	583	84.6	398	81.8	799	86.8
Non-conventional^b^	104	14.5	106	15.4	89	18.2	121	13.2
**Education **(n)	(1423)	NS	(1422)	NS				
No^c^	37	5.1	54	7.7	34	7	57	6.1
Low^d^	185	25.5	199	28.6	145	29.6	239	25.7
Inter^e^	399	54.9	361	51.8	252	51.4	508	54.5
High^f^	105	114.5	83	11.9	59	12	128	13.7
**Occupational status **(n)	(1381)		NS		(1380)		χ^2^(3) = 36.9, p < 0.0001	
Blue-collar worker	403	57.4	395	58.2	300	64.1	498	54.6
Low white-collar worker	35	5	30	4.4	23	4.9	42	4.6
Middle/high white-collar worker	82	11.7	71	10.4	67	14.3	86	9.4
Student/other	182	25.9	183	27	78	16.7	286	31.4
**Socio-economic status** (n)	(1417)		NS		(1416)		χ^2^(3) = 39.4, p < 0.0001	
Work for others^g^	264	36.4	201	29	197	40.2	268	29
Liberal/own business^h^	118	16.3	130	18.8	87	17.8	161	17.4
Student	184	25.3	182	26.3	80	16.3	285	30.8
* Domestic/others*^*i*^	159	22	179	25.9	126	25.7	212	22.8
**Salary/financial resources **(n)	(1386)		NS		(1386)		χ^2^(1) = 16.1, p = 0.0001	
Yes	344	48.4	293	43.4	256	53.3	381	42.1
**Financial strain **(n)	(1405)		NS		(1404)		χ^2^(1) = 6.3, p = .012	
Yes	605	84.1	589	85.7	429	88.3	764	83.2
**BMI** (n)	(1316)		NS		(1315)		(F(1,1313) = 10.7, p = 0.0011	
Mean ± SD	24.9 ± 4.5		24.7 ± 4.9		25.4 ± 4.7		24.5 ± 4.7	
**Alcohol use **(n)	(1412)		χ^2^(1) = 15.1, p < 0.0001		(1411)		χ^2^(1) = 9, p = 0.0026	
Yes	300	41.7	220	31.7	205	42.2	315	34.1
**Cigarette use **(n)	(1399)		χ^2^(1) = 6.6, p = 0.011		(1398)		χ^2^(1) = 23.8, p < 0.0001	
Yes	62	8.7	36	5.2	56	11.6	42	4.6

^a^= e.g. own/rent housing in cement; ^b ^= e.g. own/rent housing in local materials wood; ^c ^= cannot read/write; ^d^ = primary school/similar

^e^= secondary school/similar; ^f ^= university/similar; ^g^ = e.g. clerks; ^h ^= e.g. accountants; ^i ^= at home/unemployed.

### Measures

Dependent variables: Intimate partner violence (IPV) was assessed with the CTS2 scales [30], from which the dependent variables (psychological aggression, physical assault, sexual coercion, physical assault with injury) were derived. The women were asked whether they perpetrated the various types of IPV (39 items) and then whether they had been victims of the same IPV types (39 items). Thus, the CTS2 has a total number of 78 items. The CTS2 covers negotiation, emotional (e.g. said I was sure we could work out a problem) and cognitive (e.g. explained my side of a disagreement to my partner); psychological aggression, severe (e.g. called my partner fat or ugly) and minor (e.g. shouted or yelled at my partner); physical assault, severe (e.g. kicked my partner) and minor (e.g. grabbed my partner); sexual coercion, severe (e.g. used threats to make my partner have sex) and minor (e.g. made my partner have sex without a condom); physical assault with injury, severe (e.g. had a broken bone from a fight with my partner) and minor (e.g. had a sprain, bruise, or small cut because of a fight with my partner); and chronicity (how often the acts happened). Frequency of the acts was categorized as once, twice, 3–5, 6–10, 11–20 or >20 times during the past year, and did not occur within the past year, but before the last year or never occurred.^b^ Chronicity was assessed by calculating the mean number of times the violent acts in each scale (minor, severe, overall) occurred among those who experienced at least one violent act [30]. The validity and reliability of CTS2 has been shown to be good [30]. Further, we measured who initiated the physical assault.^c^ In this study, the questions on negotiation and who initiated the physical assault were not processed. Detailed bivariate data on perpetration are not presented here. The Cronbach α´s were 0.82 for psychological aggression, 0.89 for physical assault, 0.73 for sexual coercion and 0.65 for physical assault with injury.

Abuse as a child was assessed with 4 open questions, one each for psychological abuse (e.g. shouted or yelled at), physical abuse (e.g. beaten up), sexual abuse (e.g. forced to have sex) and injury (e.g. bruised). Additionally, chronicity (how often the acts happened) was assessed. Frequency of the acts was assessed as: once, twice, 3–5, 6–10, 11–20 or >20 times or never happened.^d^ The questions provided information about the respondent’s exposure to abuse before the age of 15 years. Detailed bivariate data on childhood abuse are not presented here. The Cronbach α´s were 0.70 for psychological abuse, 0.72 for physical abuse, 0.68 for sexual abuse and 0.71 for injury.

Controlling behaviours were assessed with the Controlling Behaviour Scale Revised (CBS-R) [31,32]. The CBS-R consists of 24 questions, does not involve questions of physical assault and has good discriminative ability [31,32]. Questions include controlling the other’s money and try to restrict time one spent with family or friends. The response format has a range of 0–4 (never to always), and a total range of 0–96. The women were required to report how often they used each act of control toward their partners in the last year and how often their partners used each act on them; high scores correspond to high control. Detailed bivariate data on controlling behaviours are not presented here. Cronbach α’s for women being controlled and using controlling behaviours were 0.93 and 0.91, respectively.

Life-style variables: (i) Use of alcohol and cigarettes (yes or no); (ii) A Body Mass Index (BMI), based on self-reported height and weight, and computed for each woman with the formula kg/m^2^. BMI was computed because evidence has shown that eating disorders (e.g., anorexia, bulimia) are among the mental health consequences of IPV. Thus, excessive weight gain or loss may be some of the first indications of such trauma [36].

Demographic/socio-economic variables included: age (in years), marital status (single, married/cohabitant, divorced/separated, widow), children at women (yes or no), housing (conventional or non-conventional), education (cannot read/write, low, intermediate, high), occupational status (blue collar, low white-collar, middle/high white-collar, student/other), socio-economic status (work for others, liberal/own business, student, domestic/other), salary/financial resources (yes or no), and financial strain i.e. concerns about how to make ends meet or provide for her needs, was assessed with one question in a no/sometimes/often/always format. A woman was defined as experiencing financial strain if she selected any response other than no. These items were largely derived from a classification system concerning socio-economics etc., which is used in Mozambique (Ministry Council–Ministry of Finances).

### Design/procedure

Data from this cross-sectional study were collected during 12 months (consecutive cases) among women who had contact with the Forensic Services at the Maputo Central Hospital (Maputo City, Mozambique) for their IPV experiences between April 1, 2007 and March 31, 2008. The women were either self-referred, referred by female organizations, or referred by the Police, with the majority being referred by female organizations or self-referred. No annotations were made concerning how many of the women were referred by female organisations and police, and self-referred. Trained female interviewers (medical students at the Faculty of Medicine and nurses at the Forensic Services) informed the women about all details of the research, what was expected of them and the way information would be handled (verbally or verbally/in writing). Strong emphasis was put on voluntariness, confidentiality and that non-participation would not lead to any negative effects. After receiving permission, the women were interviewed (on average 1 hour) in a private room by means of a questionnaire. Data management (e.g. processing) were conducted according to usual anonymous and confidentiality rules, and findings from the study would be published, using aggregated and de-identified data only. Feed-back information on the study will be made available to participants, at request, as aggregate data relationships. The National Ethical Committee at the Ministry of Health of Mozambique approved the study.

### Statistical analyses

Data on IPV types (psychological aggression, physical assault, sexual coercion, physical assault with injury) during the past 12 months were described in form of raw figures, percentages, means and standard deviation (SD). The relation between IPV types, demographic/socio-economic and life-style variables were examined with analysis of variance (ANOVAs) and chi-square tests (χ^2^). The significance level for bivariate analyses was set at p < 0.0125 and for multivariate analyses at p < 0.05.

Four multiple block-wise logistic regressions were conducted to identify and quantify factors associated with IPV types (e.g. physical assault) during the past 12 months, while controlling for other possible factors. In block-wise logistic regression, variables are entered into the regression equation block by block and the contribution of each block in explaining the dependent variable is expressed as Nagelkerke R^2^. Each block explains part of the total variance (total model). Nagelkerke R^2^ is an approximation to descriptive goodness-of-fit statistics to assess the fit of the proposed logistic model (quantify the strength of association between variables) [37]. Results were expressed in form of odds ratios (ORs) and 95% confidence intervals (95% CI).

In the regressions, the independent factors included were variables significantly related to each IPV type in the bivariate analyses. Demographics/socio-economics were entered first in the models followed by life-style, controlling behaviours, perpetration, victimisation and abuse as a child, a procedure often used in studies in the field. Concretely, the included independent factors were marital status, having children at home, housing, education, occupational status, socio-economic status, having salary/financial resources, financial strain, BMI, and smoking and alcohol use (yes/no). Further, we added controlling behaviours over/by partner, women’s IPV perpetration and victimisation by type (e.g. psychological aggression)^e^ and childhood abuse (e.g. psychological). The regression models differed for each IPV type depending on results of the bivariate analyses, but some of the variables (e.g. control) were used in all models. All variables with more than two categories were transformed into dummy variables.

## Results

### Occurrence, chronicity, severity and co-occurrence of IPV

As shown in Table 2, 45.9% (chronicity, 11.4 ± 19.8)^f^ of the women experienced severe psychological aggression; 44% (chronicity, 12.8 ± 26.9) severe physical assault; 29.2% (mean chronicity, 6.1 ± 15.1) severe sexual coercion; and 26.3% severe physical assault with injury (chronicity, 3.1 ± 9.1). The overall experienced IPV across severity (one or more types, minor and severe) was 70.2% (chronicity, 85.8 ± 120.9), of which 55.3% was severe abuse (chronicity, 33.4 ± 58.3). Further, co-occurring IPV exposure across severity showed the following patterns: 26.8% (chronicity, 55.3 ± 117.6) of the women experienced all IPV types; 31.8% (chronicity, 52.4 ± 101.3) experienced a combination of psychological aggression, physical assault and physical assault with injury; 27.1% (chronicity, 35.7 ± 80.3) experienced a combination of physical assault, sexual coercion and physical assault with injury; 42.6% (chronicity, 64.9 ± 110.9) experienced a combination of psychological aggression, physical assault and sexual coercion; and 27.4% (chronicity, 36.4 ± 74.0) experienced a combination of psychological aggression, sexual coercion and physical assault with injury.

**Table 2:** The distribution of IPV by single and co-occurring types and chronicity during the past 12 months among women

**Variables**	**Victim n = 1433**
**Psychological aggression **(n)	
**Minor**	913
Occurrence (Percentage)	63.7
Chronicity, Mean ± SD	23.9 ± 29.8
**Severe**	658
Occurrence (Percentage)	45.9
Chronicity, Mean ± SD	11.4 ± 19.8
**Total**^**a**^	936
Occurrence (Percentage)	65.3
Chronicity, Mean ± SD	35.3 ± 45.5
**Physical assault **(n)	(1432)
**Minor**	729
Occurrence (Percentage)	50.9
Chronicity, Mean ± SD	14.9 ± 27.6
**Severe**	629
Occurrence (Percentage)	44
Chronicity, Mean ± SD	12.8 ± 26.9
**Total**^**a**^	778
Occurrence (Percentage)	54.3
Chronicity, Mean ± SD	27.7 ± 51.5
**Sexual coercion **(n)	
**Minor**	686
Occurrence (Percentage)	47.9
Chronicity, Mean ± SD	10.2 ± 16.4
**Severe**	418
Occurrence (Percentage)	29.2
Chronicity, Mean ± SD	6.1 ± 15.1
**Total**^**a**^	730
Occurrence (Percentage)	50.9
Chronicity, Mean ± SD	16.3 ± 28.3
**Physical assault with injury **(n)	(1432)
**Minor**	432
Occurrence (Percentage)	30.2
Chronicity, Mean ± SD	3.3 ± 8.9
**Severe**	377
Occurrence (Percentage)	26.3
Chronicity, Mean ± SD	3.1 ± 9.1
**Total**^**a**^	492
Occurrence (Percentage)	34.4
Chronicity, Mean ± SD	6.4 ± 15.4
**Overall IPV**^**b**^	
**Minor**	987
Occurrence (Percentage)	68.9
Chronicity, Mean ± SD	52.4 ± 67.8
**Severe**	793
Occurrence (Percentage)	55.3
Chronicity, Mean ± SD	33.4 ± 58.3
**Total**	1006
Occurrence (Percentage)	70.2
Chronicity, Mean ± SD	85.8 ± 120.9
**Variables**	Victim **n = 1433**
**Co-occurrence of IPV **(n)	
**All types**^**c**^	384
Occurrence (Percentage)	26.8
Chronicity, Mean ± SD	55.3 ± 117.6
**Three types**^**d**^	456
Occurrence (Percentage)	31.8
Chronicity, Mean ± SD	52.4 ± 101.3
**Three types**^**e**^	388
Occurrence (Percentage)	27.1
Chronicity, Mean ± SD	35.7 ± 80.3
**Three types**^**f**^	610
Occurrence (Percentage)	42.6
Chronicity, Mean ± SD	64.9 ± 110.9
**Three types**^**g**^	393
Occurrence (Percentage)	27.4
Chronicity, Mean ± SD	36.4 ± 74.0

^a^= exposed to minor and severe IPV; ^b ^= exposed to minor, severe IPV (one or more types); ^c ^= all types (psychological aggression, physical assault, sexual coercion and physical assault with injury across severity); ^d ^= psychological aggression, physical assault and physical assault with injury across severity; ^e^= physical assault, sexual coercion and physical assault with injury across severity; ^f ^= psychological aggression, physical assault and sexual coercion across severity; ^g ^= psychological aggression, sexual assault and physical assault with injury across severity.

### Demographics/socio-economics, life-style and IPV

Psychological aggression. As shown in Table 1, psychologically aggressed women compared to counterparts more often were single, more often had secondary education levels and more often used alcohol. Physical assault. Physically assaulted women compared to counterparts more often were single and more often used alcohol. Sexual coercion. Sexually coerced women more often were single and more often used alcohol/cigarettes. Physical assault with injury. Injured women compared to counterparts more often were single and blue-collar workers, more often worked for others and resided in conventional housing, more often had salary/financial resources and children at home, and experienced greater financial strain. They also had a higher BMI and more often used alcohol/cigarettes.

### Factors associated with IPV

As shown in Table 3, the likelihood of psychological aggression was lower among divorced/separated women (OR = 0.34; 95% CI = 0.17-0.69) compared to single women, and women who were controlled by partner (OR = 0.98; 95% CI = 0.97-0.99) compared women who were not controlled. In contrast, the likelihood of psychological aggression was higher among women with middle (OR = 3.17; 95% CI = 1.31- 7.64) and high education (OR = 3.33; 95 CI = 1.27-8.73) compared to women who could not read or write. Women who controlled their partner (OR = 1.03; 95% CI = 1.01-1.05) were more likely to experience psychological aggression. In addition, the likelihood of being victimised was higher among women who perpetrated physical assault against male partner (OR = 2.40; 95% CI = 1.22-4.74) compared to those who did not, and among women who experienced co-occurrence of victimization [physical assault & psychological aggression (OR = 13.63; 95% CI = 1.65-24.29); sexual coercion & psychological aggression (OR = 8.23; 95% CI = 4.69-14.44); and physical assault with injury & psychological aggression (OR = 4.25; 95% CI = 2.01-8.98), compared to those who did not experience co-occurring victimization. Women who were physically abused as a child (OR = 1.51; 95% CI = 1.01-2.25) were more likely to experience psychological aggression compared to those who did not.

**Table 3:** Variables associated with IPV by type among women during the 12 months prior to the survey

**Dependent variables**	**Psychological aggression**^**m**^	**Physical assault**^**m**^	**Sexual coercion**^**m**^	**Physical assault with injury**^**m**^ **N = 1040**
	**N = 1265**	**N = 1271**	**N = 1258**	
	**OR 95% CI**	**OR 95% CI**	**OR 95% CI**	**OR 95% CI**
**Independent variables**				
**Demographics/socio-economics**				
**(Block 1)**				
Marital status^a^				
Married/cohabitant	1.28 (0.82–2.01)	1.19 (0.77–1.85)	0.80 (0.55–1.15)	1.07 (0.66–1.72)
Divorced/Separated	0.34 (0.17–0.69)	0.99 (0.45–2.17)	0.40 (0.22–0.71)	1.88 (0.92–3.82)
Widow	0.65 (0.28–1.51)	1.52 (0.61–3.82)	0.46 (0.21–1.03)	0.85 (0.33–2.17)
Single^c^	1	1	1	1
Children at home^a^	NA^1^	NA^1^	NA^1^	
Yes				2.69 (1.51–4.79)
No^c^				1
Housing^a^	NA^1^	NA^1^	NA^1^	
Non-conventional^d^				1.12 (0.58–2.16)
Conventional^c, e^				1
Education^a^		NA^1^	NA^1^	NA^1^
Low^f^	2.32 (0.92–5.83)			
Middle^g^	3.17 (1.31–7.64)			
High^h^	3.33 (1.27–8.73)			
Cannot read/write^c^	1			
Occupational status^a^	NA^1^	NA^1^	NA^1^	
Blue-collar worker				0.82 (0.14–4.75)
Low white-collar worker				0.66 (0.09–4.86)
Inter/high white-collar worker				0.94 (0.14–6.23)
Student/other^c^				1
**Socio-economic status**^**a**^	NA^1^	NA^1^	NA^1^	
Work for others^i^				0.60 (0.11–3.28)
Liberal/own business^j^				0.85 (0.15–4.78)
Domestic/other^k^				1.70 (0.32–9.11)
Student^c^				1
Salary/financial resources^a^	NA^1^	NA^1^	NA^1^	
Yes				1.70 (0.92–3.16)
No^c^				1
Financial strain^a^	NA^1^	NA^1^	NA^1^	
Yes				1.44 (0.83–2.49)
No^c^				1
R^2^^n^	(7.1)	(2.1)	(3.4)	(7.5)
**Life-style**				
**(Block 2)**				
BMI^b^	NA^1^	NA^1^	NA^1^	1.01 (0.97 1.06)
Drinking^a^				
Yes	0.89 (0.60–1.33)	0.93 (0.62–1.40)	1.33 (0.95–1.87)	0.73 (0.49–1.10)
N0^c^	1	1	1	1
Smoking^a^	NA^1^	NA^1^		
Yes			1.43 (0.65–3.14)	2.22 (0.92–5.33)
No^c^			1	1
R^2^^n^	(0.08)	(1)	(2.1)	(2.3)
**Controlling behaviours over partner**				
**(Block 3)**				
Control^b^	1.03 (1.01–1.05)	1.00 (0.98–1.02)	1.02 (1.00–1.03)	1.00 (0.98–1.02)
R^2^^n^	(12.8)	(16.2)	(15)	(12.6)
**Controlling behaviours by partner**				
**(Block 4)**				
Control^b^	0.98 (0.97–0.99)	1.03 (1.02–1.04)	1.00 (0.99–1.01)	1.04 (1.03–1.05)
R^2^^n^	(0.05)	(4.1)	(1)	(6.6)
**Perpetration**				
**(Block 5)**				
Psychological aggression^a^	NA^1^			
Yes		4.04 (2.22–7.33)	1.89 (1.10–3.27)	1.95 (0.86–4.42)
No^c^		1	1	1
Physical assault^a^		NA^1^		
Yes	2.40 (1.22–4.74)		1.31 (0.88–1.94)	1.39 (0.89–2.16)
No^c^	1		1	1
Sexual coercion^a^			NA^1^	
Yes	0.95 (0.53–1.71)	0.93 (0.57–1.52)		1.95 (1.22–3.11)
No^c^	1	1		
Physical assault with injury^a^				NA^1^
Yes	0.89 (0.33–2.40)	3.56 (1.55–8.15)	2.02 (1.22–3.32)	
No^c^	1	1	1	
R^2^^n^	(29.8)	(42.5)	(31.5)	(25.3)
**Victimization**				
**(Block 6)**				
Psychological aggression^a^	NA^1^			
Yes		8.37 (4.36–16.06)	5.80 (3.24–10.39)	2.81 (1.08–7.35)
No^c^		1	1	1
Physical assault^a^		NA^1^		
Yes	13.63 (1.65–24.29)		3.71 (2.41–5.70)	10.61 (5.21–21.61)
No^c^	1		1	1
Sexual coercion^a^			NA^1^	
Yes	8.23 (4.69–14.44)	4.33 (2.70–6.95)		1.14 (0.68–1.93)
No^c^	1	1		1
Physical assault with injury^a^				NA^1^
Yes	4.25 (2.01–8.98)	7.54 (4.21–13.53)	1.11 (0.72–1.71)	
No^c^	1	1	1	
R^2^^n^	(19.9)	(9.2)	(6.5)	(6.2)
**Abuse as a child**				
**(Block 7)**				
Psychological abuse^a^				
Yes	1.40 (0.51–3.84)	0.94 (0.40–2.24)	1.56 (0.68–3.57)	0.42 (0.18–1.02)
No^c^	1	1	1	1
Physical abuse^a^				
Yes	1.51 (1.01–2.25)	0.91 (0.60–1.36)	0.92 (0.65–1.28)	0.69 (0.46–1.04)
No^c^	1	1	1	1
Sexual abuse^a^				
Yes	0.84 (0.30–2.34)	0.59 (0.22–1.64)	1.54 (0.66–3.57)	2.28 (0.99–5.21)
No^c^	1	1	1	1
Injury^a^				
Yes	0.53 (0.22–1.27)	1.29 (0.58–2.88)	1.71 (0.86–3.41)	1.57 (0.80–3.07)
No^c^	1	1	1	1
R^2^^n^	(0.03)	(0.01)	(0.03)	(0.09)
Total R^2^	(69.76)	(75.11)	(59.53)	(60.59)

^a^= category variables; ^b ^= continuous variables; ^c^ = comparison categories; ^d ^= e.g. own/rent housing in local materials such as wood; ^e ^= e.g. own/rent housing in cement; ^f ^= primary school/similar.

^g^= secondary school/similar; ^h ^= university/similar; ^i^ = e.g. clerks; ^j ^= e.g. accountants; ^k ^= at home/unemployed; ^l ^= NA, non-applicable; ^m ^= different models are used in terms of independent variables; ^n ^= proportion of the total R^2^ explained by each block; R^2 ^= Nagelkerke; *OR*, Odds Ratio; 95% CI = 95% Confidence interval.

Physical assault was more likely among who were controlled by partner (OR = 1.03; 95% CI = 1.02-1.04), among women who perpetrated psychological aggression (OR = 4.04; 95% CI = 2.22-7.33) compared to those who did not, among women who perpetrated physical assault with injury (OR = 3.56; 95% CI = 1.55-8.15), among women who experienced co-occurring victimization [psychological aggression & physical assault (OR = 8.37; 95% CI = 4.36-16.06), among women who experienced co-occurring sexual coercion & physical assault (OR = 4.33; 95% CI = 2.70-6.95), and among women who experienced co-occurring physical assault with injury & physical assault (OR = 7.54; 95% CI = 4.21-13.53) compared to women who did not.

The likelihood of being sexually coerced was lower among divorced and separated women (OR = 0.40; 95% CI = 0.22-0.71) compared to those who were single. Sexual coercion was more likely among women who perpetrated psychological aggression (OR = 1.89; 95% CI = 1.10-3.27) and physical assault with injury (OR = 2.02; 95% CI = 1.22-3.32), among women who experienced co-occurring psychological aggression & sexual coercion (OR = 5.80; 95% CI = 3.24-10.39), and physical assault & sexual coercion (OR = 3.71; 95% CI = 2.41-5.70) compared to women who did not.

Physical assault with injury was more likely among women who had children at home (OR = 2.69; 95% CI = 1.51-4.79) compared to those who did not have children at home, as well as by women who were controlled by partner (OR = 1.04; 95% CI = 1.03-1.05) compared to those who were not. Women who perpetrated sexual coercion (OR = 1.95; 95% CI = 1.22-3.11) were more likely to experience physical assault with injury compared to those who did not perpetrate abuse. In addition, women who experienced co-occurring victimization [psychological aggression & physical assault with injury (OR = 2.81; 95% CI = 1.08-7.35), and physical assault & physical assault with injury (OR = 10.61; 95% CI = 5.21-21.61) compared to those who did not experience co-occurring victimization.

## Discussion

This study of women who contacted the Forensic Services at the Maputo Central Hospital for abuse assessed women’s experiences of IPV by type during the preceding 12 months, examined the association between IPV types and predictor variables.

### Occurrence, chronicity and severity of IPV

This cross-sectional study showed that majority (70.2%, chronicity, 85.8 ± 120.9) of the women had experienced one or more IPV types during the past 12 months, of which more than half the number (55.3%, chronicity, 33.4 ± 58.3) of cases were severe acts., The overall figures in this study were higher than those reported in SSA and elsewhere with different population samples, which varies between 2 - 51% [1,2,4-7,14,38,39]. Variation in the operationalization of IPV, collecting data procedures and characteristics of the samples may explain differences in abuse rates. The chronicity and severity findings in our study are not comparable with those of other studies, given that such data are either not provided or are reported differently.

The most common form of IPV was psychological aggression, 65.3%; chronicity, 35.3 ± 45.5), with severe acts amounting to 45.9% (chronicity, 11.4 ± 19.8). Our findings are higher than those observed in SSA and elsewhere with various types of populations, which range from 18.2–51% [6,10,13,14,38,40], and slightly lower than those reported in a Russian study with female students using the same operational definition of psychological aggression (66.7%) [41]. The chronicity and severity rates in the present study are however higher than those reported in this Russian study using the same operational definitions (4.3%; mean chronicity of 13) [41]. These differences may be related to these factors being more common/salient in women seeking help for abuse by partner than amongst students.

Our finding that physical assault was experienced by 54.3% (chronicity, 27.7 ± 51.5) of the women, with 44% (chronicity, 12.8 ± 26.9) being severe acts, are greater than those shown in SSA and elsewhere (5–38%) [1,6,9,14,38,41,42], and in a study from Mozambique (11%) [25] that addressed a limited number of acts of physical assault. In contrast, a study of physical assault in Canada showed higher rates of physical assault than those reported by women in the present study (67% and 68% of women were exposed to minor and severe physical assault, respectively) [43]. Discrepancies in abuse rates could reflect differences in the operational definition of physical assault, types of participants and how the data are collected, although the figures from Canada suggest that factors other than sample characteristics and culture may be more important, requiring further investigation.

Sexual coercion was reported by 50.9% (chronicity, 16.3 ± 28.3) of the women, and 29.2% (chronicity, 6.1 ± 15.1) were severe acts. Our figures are higher than those observed in Sub-Saharan Africa and elsewhere among different samples, which range from 5.2–44.4% [5-7,9,11,38,41,42,44]. In this study the willingness of our respondents to disclose their experiences of sexual abuse may explain differences in rates

Physical assault with injury was reported by 34.4% (chronicity, 6.4 ± 15.4) of the women, and 26.3% (chronicity, 3.1 ± 9.1) were severe acts. Our rates are consistent with those of previous observations, although in some cases in the upper range, in SSA and elsewhere (e.g. Canada) with different samples (e.g. community), which vary between 9 - 45.9% [8,10,43], suggesting that the sample type may not have a major importance in the occurrence of physical assault with injury. Similarly to the other forms of abuse, our results concerning the chronicity and severity of injuries may be difficult to compare with those of other studies due to a lack of such data or shown differently (e.g. chronicity, 0 times, 1–2 times, 3–5 times, >5 times). However, our figures are greater than those shown in a Russian study with female students using the same operational definition of chronicity and severity (7.7% were severe acts at a mean chronicity of 1.2) [41].

### Co-occurrence and chronicity of IPV

Co-occurring victimization across severity was common, with 26.8% (chronicity, 55.3 ± 117.6) of the women experiencing all IPV types. The rates of victimisation regarding the other composite forms of IPV across severity (4 combinations with 3 types in each) varied between 27.1 - 42.6% and chronicity between 35.7 ± 80.3 - 64.9 ± 110.9, depending on the combination. The combination psychological aggression, physical assault and physical assault with injury had the highest figures contrasted to the other combinations. In this study rates of co-occurring IPV, in case combinations are similar, compared to those reported in Sub-Saharan Africa and elsewhere are higher [5,38] and lower [45,46]. For example, in a review of IPV in 7 African countries among general population samples (Cameron, Kenya, Malawi, Rwanda, Uganda, Zambia, and Zimbabwe), the rates of co-occurring emotional aggression, physical assault and sexual coercion varied between 3.6-8.3% [4], whereas our rate was 27.4%. On the other hand, a study from Japan with a sample of convenience reported a much higher figure for the emotional aggression, physical assault and sexual coercion constellation, i.e. 57% [46]. The differences in the findings concerning women´s experience of all IPV types are not comparable to those of other studies, reasons most likely being methodological as well as differences in social context. Our figures on the chronicity and severity of the composite abuse are difficult to contrast with those of other studies as they either do not present such data or report differently (e.g. chronicity, never to all the time). Discrepancies in the co-occurring abuse rates may be due to several factors such as differences in how co-occurring abuse is combined, the operational definition of IPV and the characteristics of the samples. Culture may not be an important factor in the co-occurrence of IPV as exemplified by our findings and those of the Japanese study [46]. In any case, we are facing a group of women with multiple and frequent experiences of IPV. We posit that the higher prevalence of IPV found among women in our study may reflect higher disclosure facilitated by the use of the ascertainment tools – the CTS2 and the CBS-R in this study, which aided in the facilitation of women’s interpretations of these abusive acts, thereby increasing disclosure of abuse.

The rates of IPV in this study tend to surpass those observed in other studies with different populations (e.g. women attending antenatal clinics), including studies from Sub-Saharan Africa, although there are exceptions concerning physical assault and physical assault with injury. Indeed, in some cases our rates of physical assault and physical assault with injury are lower than those reported in general population samples. In several instances, differences in abuse rates may be partly explained by discrepancies in the operational definition of IPV (as evidenced by the increased disclosure when using ascertainment tools in this case of this study), the number of violence items assessed and the population characteristics (women who sought help for IPV experiences). However, the issue of culture was not addressed here. Other explanations (as shown in the regressions) could be that the women were also abusive. Reciprocal abuse was common and controlling behaviours was an important component in the abuse. The data on the chronicity and severity of abuse are difficult to compare with other observations due to a lack of such information or reported differently, but our figures were higher than those shown in a study using the same operational definition of IPV, chronicity and severity. In any case, our findings point for instance to the necessity of using the same operational definition of IPV, chronicity and severity when doing research about abuse against women as well as the need of further research across cultures and types of respondents.

### Differences in demographics/socio-economics/life-style and correlates of IPV

As shown in the bivariate analyses, single women more often experienced all types of abuse during the past 12 months, women with Intermediate level education more often experienced psychological aggression, as well as women who had children at home, lived in conventional housing, were blue collar workers, worked for others, had salary and experienced financial strain were more likely to experience physical assault with injury. These findings are in line with those from other studies indicating increased likelihood of abuse among women of low socio-economic status due to their limited resources, economic vulnerability and limited opportunities [1,2,6,8,25]. The findings that women who used alcohol more often experienced all types of abuse, and women who used cigarettes more often experienced sexual coercion and physical assault with injury are in line with those from previous studies [8,12,26]. This may be attributed to alcohol consumption and intoxication leading to irresponsible behaviour, thus increasing the likelihood of violence.

Results of the logistic regression analyses revealed that of the demographic, socio-economic and life-style variables, only middle/high education levels, divorce/separation and having children at home were independently associated with experienced IPV, in addition to controlling behaviours over/by partners, own perpetration, co-occurring victimization and abuse as a child.

The finding that higher educational levels were positively associated with exposure to psychological aggression may be associated with the notion that in Mozambique, the empowerment conferred by better education may have been insufficient to counteract traditional gender roles; rather, women’s higher education tends be associated with increased vulnerability to violence. It is also possible that the more educated and empowered the women were, the more assertive they became about their rights, being less inclined to accept traditional cultural and gender roles in which women are expected to be submissive to men, thus triggering abuse. Our results are in contrast with studies in SSA and elsewhere (e.g. USA), which report no effects of education on IPV/reduced IPV risk among high educated women [13,38,44,47-49] or an increased IPV risk among low educated women [8,50,51].

Divorce/separation was negatively associated with experienced psychological aggression/sexual coercion in this study. One could hypothesize that by terminating the relationship the women reduced the likelihood of further violence, which is in agreement with findings from a study in Malawi with a general population sample indicating that IPV is a contributing factor for divorce/separation [13]. Available data on this issue with various types of respondents (e.g. women who had survived an attempted homicide and controls), often about physical assault/sexual coercion, in SSA and elsewhere are otherwise conflicting, with some studies reporting that divorce/separation is connected with increased IPV risk [27,38,48,52-54] and others not finding such connections [8,55].

Having children at home was positively associated with the experience of physical assault with injury. Though it is not possible to draw causal inference between number of children at home and the likelihood of abuse due to the cross-sectional design of this study, the absence of an association between age and violence suggests that violence may also have begun before some women have started bearing children, which is consistent with findings from other studies [51,55]. This may also be related to the increased economic stress associated with increasing number of children, as indicated by other studies which indicate increased likelihood of domestic violence associated with increasing financial strain [56]. However, caution needs to be exercised when interpreting this finding, given that the relationship may also be reciprocal in nature i.e. domestic violence may also cause financial problems for victims of domestic violence, thus entrapping them in poverty and an abusive relationship [57]. Studies have also shown that individuals are more reluctant to leave abusive relationships when they have investments such as children in the relationship, emotional attachment etc. [58]. Other studies from SSA and elsewhere (e.g. Spain) have produced conflicting results, with some showing positive [42,44,48,50] and some showing negative results [49,55].

Controlling behaviours over partners were positively associated with exposure to psychological aggression/sexual coercion. No previous study in Mozambique has addressed the effect of women’s control over partners on their own victimization. It is possible that in Mozambique, where traditional gender roles seem to remain strong, women’s control over partners is not accepted resulting in increased abuse vulnerability. Other explanations could be that women used control to counter dominant partners, reduce violence, attain something blocked by dominant partners or change the power structure in the relationship, and thereby increased their abuse vulnerability rather than the opposite. In any case, our findings are in line with a study involving physical assault from Ghana with university students using the same operational definition of controlling behaviours [19], which showed that female and male students used a similar frequency of controlling behaviours victimization/perpetration, and that controlling behaviours “predicted” IPV. Studies from the United Kingdom (UK) with different samples (e.g. battered women) partly using the same operational definition, mostly about physical assault/sexual coercion, have also reported a relation between controlling behaviours and victimization/perpetration among both sexes [31,41,59].

In addition, controlling behaviours by partners were positively associated with the experience of physical assault/physical assault with injury, and negatively with psychological aggression. Our findings corroborate those from previous studies from SSA, which indicate that male control is associated with increased IPV vulnerability for women [2,5,14-16]. Furthermore, given the gender inequalities in Mozambique, as in other low- and middle-income countries with existing patriarchal norms and values, women are more accepting of prevalent gender power structure within. This makes them less assertive, more vulnerable, and more dependent on their male partners for material needs, thereby increasing their likelihood of experiencing IPV [60]. Overall, our findings indicate some degree of reciprocity in terms of controlling behaviours and its relationship with IPV, as observed in relation to physical assault/sexual coercion in investigations from the UK) with for instance battered women [31,41,59], and in Ghana with university students [19] using partly the same/same operational definition. Our results thus suggest that controlling behaviours may be more significant to the occurrence of victimisation/perpetration than sample characteristics and culture.

### Women’s perpetration of abuse

Women’s abuse of their partners (physical assault, psychological aggression, sexual coercion, physical assault with injury) was positively associated with their own victimization (physical assault, psychological aggression, sexual coercion, physical assault with injury). As far as we know, our study is the first in SSA (including Mozambique) to address the influence of women’s perpetration on their own victimization. It is plausible that women’s perpetration of IPV may have been a defensive reaction to their partners aggression, retribution to it or as a kind of preventive measure to avoid future aggression, but resulted in increased abuse vulnerability. This pattern however indicates that the women may have been involved in relationships where mutual aggression may be common. Our results are in line, for example, with studies of women selected to represent victims of male IPV from USA, mostly about physical assault, reporting that women use violence against their male partners [31,61]. While some authors suggested that women’s partner violence happens as a reaction/defence to men’s violence [62-64], others have observed that where one partner is the sole perpetrator, it is not uncommon for this individual to be a woman than a man [65], with higher incidence in younger women [59]. Interestingly, various studies from SSA with different types of respondents (e.g. outpatients presenting to prenatal and paediatric clinics), mostly about physical assault/sexual coercion, have reported that women may also initiate and abuse their male partners [10,12-15,26,28].

We found that the different IPV types tended to co-occur. Whether one form of IPV is triggering off to another or occurred more or less simultaneously was not ascertained, but the women were involved in highly violent relationships. While it is unclear why women endured such relationships, we postulate that part of the explanation may be denial, fear, self-blame, lack of support and/or inability to challenge the cultural norms that may tolerate violence against women [34]. Apparently, as indicated by the regressions, socio-economic conditions and life-style did not play a major role. On the other hand, women also used IPV suggesting some degree of mutuality in their violent relationships. In any case, our data are in line with other observations from Sub-Saharan Africa and elsewhere (e.g. USA) with different samples (e.g. help seeking women) showing that different IPV forms often co-occur [1,2,5,38,55].

Of all types of victimization during childhood, only physical abuse was positively associated with exposure to IPV during adulthood i.e. psychological aggression. This is in line with findings from other studies with various samples that show a higher risk of IPV during adulthood among victims of child abuse [8,24,66-69], and may be attributed to the acceptance of violence as a norm among women who were abused in childhood. The women would have learned to conform to societal views of power imbalances within relationships, and are prone to increased likelihood of exposure to violence in intimate relationship. The women could also have a tendency to select partners that are consistent with the distorted views within the context in which they live.

### Strengths and limitations

This study has several limitations. First, the use of data involving consecutive cases i.e. all women visited the Forensic Services at the Maputo Central Hospital for abuse by partner during the period of data collection may be a source of selection bias. In addition, the use of ascertainment tools (the CTS2 and the CBS-R) resulted in ascertainment basis. Second, the study could not establish firm causal links due to its cross-sectional design, which requires another type of design (e.g. longitudinal repeated-measures design). Third, the women had previous experiences of IPV and a comparison group (e.g. general population) was not included; as such, the sample may not be representative of women in Maputo City as well as in the rest of the country and their experiences of IPV may differ or be similar to those of women in general. On the other hand, some of our results seem congruent with other investigations in the area using different samples (e.g. general population, battered women). Fourth, basing the study on the women’s self-reported account of their IPV experiences when they made contact with the Forensic Services without the use of hospital records may have resulted in reporting bias. Fifth, the measurement of certain variables (e.g. alcohol use, BMI) in a yes/no format may have been too crude to fully capture their association with IPV. Sixth, although great attention was directed towards the translations and back-translations of the instruments as well as their cultural adaptation, it is possible that errors were made. Finally, the lack of accurate and current statistics on the general prevalence of alcohol and cigarette use in Maputo City or Mozambique precludes us from further assessing possible similarities or differences in these and other socio-demographic characteristics between our study sample and the general population. Despite these weaknesses, the strengths of this study include its confirmation of findings from previous studies and its provision of new insights when designing intervention and prevention measures.

## Conclusions

The chronicity and frequency of severe sustained IPV among women in our sample as well as the amount of co-occurring abuse were remarkable. Controlling behaviours, own perpetration, co-occurring victimization, and some extent of childhood, having better education and children at home were more important factors in “explaining” experienced IPV than demographic/socio-economic and life styles variables.

The broad and complex spectrum of these women’s problems should be a cause of great concern for many groups (e.g. policy makers, health care providers). Urgent attention is required as their problems are likely to have profound negative consequences for themselves, others and society. In view of our findings, the urgent development of prevention and treatment interventions is necessary. Such approaches should not only consider the women’s problems, but also both partners’ conflict-related behaviours. Additionally, considering the complexity of the problems that these women and their partners seem to face, interventions are likely to require for instance different approaches and varying time frames, and conducted by various types of professionals. Finally, more research into; for example, the influence of women’s use of control and perpetration on their victimisation is warrant, not least in a Sub-Saharan context.

### Policy implications and recommendations

Findings in this study have policy implications, among which are that the findings might be useful for changing advocacy and legal guidelines concerning IPV, might be valuable for the development of prevention and conduct interventions that consider both partners conflict-related behaviours, and might encourage further research regarding, for instance the association between women’s use of control, violence and co-occurring violence upon partners and own perpetration, not least in the context of Sub-Saharan Africa.

## Endnotes

^a^In all cases of chronicity, mean/SD

^b^The CTS2 are scored by adding the midpoints of the response categories. For the categories 0, 1 and 2 the midpoints are the same. For category 3 (3–5 times) the midpoint is 4; for category 4 (6–10 times) the midpoint is 8; for category 5 (11–20 times) the midpoint is 15; and for category 6 (>20 times) the midpoint is 25.

^c^If you have been physically assaulted by your partner or you physically assaulted your partner, who did it first. Your partner assaulted you first, you assaulted your partner first or it never happened.

^d^The “scaling” was based on CTS2.

^e^Due to multicollinearity, abuse as a perpetrator (e.g. physical assault) was not used as an independent factor for physical assault as a victim and so forth. The same procedure was used for women as perpetrators.

^f^In all cases of chronicity, mean/SD.

## Competing interest

The authors declare that they have no competing interest.

## Authors’ contributions

AEZ: Major role in study conception and coordination, collected data, performed statistical analysis and writing the manuscript. GM: participated in discussion and manuscript writing. LS: participated in discussion and manuscript writing. JJFS: participated in the design of the study, statistical analysis and manuscript writing. DA: participated in discussion, coordination and manuscript writing. All authors read and approved the final manuscript.

## Pre-publication history

The pre-publication history for this paper can be accessed here:

http://www.biomedcentral.com/1472-698X/12/35/prepub
